# Computer simulations and models for the performance characteristics of spectrally equivalent X-ray beams in medical diagnostic radiology

**DOI:** 10.4103/0971-6203.37483

**Published:** 2007

**Authors:** Akintunde A. Okunade

**Affiliations:** Department of Physics, Obafemi Awolowo University 220005, Ile-Ife Osun, Nigeria

**Keywords:** Contrast, elemental filters, medical diagnostic radiology

## Abstract

In order to achieve uniformity in radiological imaging, it is recommended that the concept of equivalence in shape (quality) and size (quantity) of clinical Xray beams should be used for carrying out the comparative evaluation of image and patient dose. When used under the same irradiation geometry, X-ray beams that are strictly or relatively equivalent in terms of shape and size will produce identical or relatively identical image quality and patient dose. Simple mathematical models and software program EQSPECT.FOR were developed for the comparative evaluation of the performance characteristics in terms of contrast (C), contrast to noise ratio (CNR) and figure-of-merit (FOM = CNR^2^/DOSE) for *spectrally* equivalent beams transmitted through filter materials referred to as conventional and k-edged. At the same value of operating potential (kVp), results show that *spectrally* equivalent beam transmitted through conventional filter with higher atomic number (*Z*-value) in comparison with that transmitted through conventional filter with lower *Z*-value resulted in the same value of C and FOM. However, in comparison with the *spectrally* equivalent beam transmitted through filter of lower *Z*-value, the beam through filter of higher *Z*-value produced higher value of CNR and DOSE at equal tube loading (mAs) and kVp. Under the condition of equivalence of spectrum, at scaled (or reduced) tube loading and same kVp, filter materials of higher *Z*-value can produce the same values of C, CNR, DOSE and FOM as filter materials of lower *Z*-value. Unlike the case of comparison of *spectrally* equivalent beam transmitted through one conventional filter and that through another conventional filter, it is not possible to derive simple mathematical formulations for the relative performance of spectrally equivalent beam transmitted through a given conventional filter material and that through kedge filter material.

The use of tube voltage (peak kilovoltage, kVp), effective energy, homogeneity coefficient and half-value layer (HVL) as measures of beam quality and quantity in medical diagnostic radiology continues to produce diversities in results obtained for image quality and patient dose. As a result of this, the use of equivalent spectral as a measure of beam quality and as a tool for the standardization of medical X-ray imaging has been reported.[1–2] Among other factors, the quality and quantity of X-ray beams for medical diagnostic purpose depend on filter material and filter thickness. The standardization of beam quality can be achieved by providing a ranking for filter materials and filter thicknesses when the technology of X-ray tube is the same. By careful choice of thickness, two different elemental filters can be made to transmit beams that are simultaneously of the same/close shape (quantity) and size (quality).[3–6] When used under the same irradiation condition, beams that are strictly equivalent or relatively equivalent in terms of shape and size will produce identical or relatively identical image quality and patient dose. The results of theoretical and experimental approaches to the comparative ranking of the transmissions of filter materials on the basis of closeness of hardening (size) and attenuation (quantity) properties have been reported earlier in literature.[7–9] Due to the differences in attenuation and absorption properties, two different elemental filters that can independently produce beams of exactly equal shape and size do not exist.

In this paper, computer simulations were carried out to obtain *spectrally* equivalent X-ray beams using elemental filters - which include those referred to as conventional, such as aluminum and copper; and k-edge, such as gadolinium and tungsten. Analytical models similar to those earlier reported[6] for quantitative parameters were derived for the evaluation of differences in parameters for image quality, such as contrast-to-noise ratio (CNR) and figure-of-merit (FOM) for filter materials that transmit *spectrally* equivalent beams. For the purpose of clinical assessment of the performance characteristics of *spectrally* equivalent beams, iterative computer software EQSPECT.FOR was developed for the evaluation of these models. Also, by least square methods, parameters which can be used for the evaluation of thicknesses of other filter materials that are ‘spectrally’ equivalent to a specified thickness of copper are presented. The definitions of some of the terms used in the computer simulations and analytical formulations that were derived are presented in Table 1.

**Table 1:** Glossary of terms used in computer simulations and mathematical formulations

T(t_m_)	The average transmittance of filter material, m at spectrally equivalent thickness of t_m_
μ_m_(E_i_),μ_w_(E_i_),μ_c_(E_i_)	Attenuation coefficient for filter material, m, water, contrast medium respectively at energy E_i_
C	Contrast (scatter-free)
C_Al_, C_Cu_	Contrast from beams filtered by aluminum and copper respectively (scatter-free)
DOSE_Al_, DOSE_Cu_, DOSE_Y_	Dose from beams filtered by aluminum, copper and yttrium respectively
DOSE_m_, DOSE_ref_	Dose from beams filtered by material, m and reference material respectively
CNR_Al_, CNR_Cu_, CNR_Y_	Contrast-to-noise ratio for beams filtered by aluminum, copper and yttrium respectively
CNR_m_, CNR_ref_	Contrast-to-noise ratio for beams filtered by material, m and reference material respectively
E_a_, E_b_	Photon energy intensity absorbed in the phosphor with and without embedded contrast medium respectively
μ_Li_/ρ	Local mass energy transfer coeficient of the phosphor at energy E_i_
μ_di_/ρ	Mass attenuation coefficient of the phosphor at energy E_i_
t_w_, t_d_, t_c_	Thickness of water phantom, phosphor and contrast medium respectively
FOM	Figure of merit
FOM_Al_, FOM_Cu_, FOM_Y_	Figure of merit for beams filtered by aluminum, copper and yttrium respectively
FOM_m_, FOM_ref_	Figure of merit for beams filtered by material, m and reference material respectively
E(t_w_)	Average energy in joules imparted to water phantom of thickness t_w_ cm by a normally incident monoenergetic photon of energy E_i_
i_max_	Maximum value of energy index
T	Transmission
T_Al_, T_Cu_, T_Y_, T_m_, T_ref_	Transmission through aluminum, copper, yttrium, material, m and reference filter material respectively
mA_m_, mA_ref_	Tube current for beams filtered by material, m and reference material respectively
mAs_m_, mAs_ref_	Tube loading for beams filtered by material, m and reference material respectively
S	Exposure time
S_Al_, S_Cu_, S_m_, S_ref_	Exposure time for beams filtered by aluminum, copper, material, m and reference material respectively
HU_m_, HU_ref_	Heat capacity for beams filtered by material, m and reference material respectively
V	Numerical value of kVp
ɸ_Al_(E_i_)	Photon fluence transmitted by aluminum filter (photon/mm^−2^)
ɸ_Cu_(E_i_)	Photon fluence transmitted by copper filter (photon/mm^−2^ )

## Theoretical formulations

In this work, T (t_m_) of filter material m at ‘spectrally’ equivalent thickness of t_m_ is given by

$$T(t_{m})\;=\;\frac{\sum_{E=1}^{V}[{ɸ}_{0}(E_{i})\exp\{-\mu_{m}(E_{i})t_{m}\}]}{\sum_{E=1}^{V}{ɸ}_{0}(E_{i})}$$

C is defined as

(2) $$C\;=\;\frac{E_{a}\;-\;E_{b}}{E_{b}}$$

E_a_ and E_b_ were calculated respectively using equations of the forms[10]

(3a) $${\text{E}}_{\text{a}}\;=\;\sum_{\text{i}}\left[\begin{array}{l} {ɸ}_{\text{0}}({\text{E}}_{\text{i}})\text{exp(}-\mu_{\text{m}}{\text{t}}_{\text{m}}-\mu_{\text{w}}{\text{t}}_{\text{p}}\text{-}\mu_{\text{c}}{\text{t}}_{\text{c}}\\ {\text{E}}_{\text{i}}\left(\frac{\mu_{\text{Li}}/\rho }{\mu_{\text{di}}/\rho }\right)\left\{1\text{ -exp}\left(-\frac{\mu_{\text{di}}}{\rho }{\text{t}}_{\text{d}}\right)\right\} \end{array}\right]$$

and

(3b) $${\text{E}}_{\text{b}}\;=\;\sum_{\text{i}}\left[\begin{array}{l} {ɸ}_{\text{0}}({\text{E}}_{\text{i}})\text{exp(}-\mu_{\text{m}}{\text{t}}_{\text{m}}-\mu_{\text{w}}{\text{t}}_{\text{p}}\text{)}\\ {\text{E}}_{\text{i}}\left(\frac{\mu_{\text{Li}}/\rho }{\mu_{\text{di}}/\rho }\right)\left\{1-\text{exp(}-\frac{\mu_{\text{di}}}{\rho }{\text{t}}_{\text{d}}\text{)}\right\} \end{array}\right]$$

CNR and FOM were respectively calculated as

(4) $$\mathrm{CNR}\;=\;\left|\frac{E_{a}-E_{b}}{\sqrt{E_{b}}}\right|$$

and

(5) $$\text{FOM}\;=\;\frac{{\text{CNR}}^{\text{2}}}{\text{DOSE}}.$$

The absorbed dose in patient (water phantom of thickness t_w_ centimeters) was determined using equation of the form[11]

(6) $$\text{DOSE}\;=\;\frac{{\text{ε(t}}_{\text{w}}\text{)}}{{\text{t}}_{\text{w}}}$$

where ε(t_w_) could be obtained using an equation of the form

(7) $$\begin{array}{l} {\text{ε(t}}_{\text{w}})=\sum_{\text{i}=\text{1}}^{\text{i}=\text{i max}}{\text{E}}_{\text{i}}\;{ɸ}_{0}{\text{(E}}_{\text{i}}\text{)exp\{}-\mu_{\text{m}}({\text{E}}_{\text{i}}){\text{t}}_{\text{m}}\}\\ \;\left[\sum_{\text{n}=\text{0}}^{\text{n}=\text{5}}{\text{β}}_{\text{n}}{\text{(E}}_{\text{i}}){\text{t}}_{\text{w}}^{\text{n}}\right] \end{array}$$

Details of the derivation of Eq. (7) can be found in the work reported by Okunade.[11]

From the earlier work reported,[6] for a given filter material m in comparison with aluminum filter at spectrally equivalent thickness,

(8) $$\begin{array}{l} \text{δ}=\frac{∆}{1+∆}\;=\;\frac{{\text{λ}}_{\text{m}}{\text{t}}_{\text{Al}}}{1+{\text{λ}}_{\text{m}}{\text{t}}_{\text{Al}}}\\ \;=\frac{{\text{S}}_{\text{Al}}-{\text{S}}_{\text{Cu}}}{{\text{S}}_{\text{Al}}}\;=\;\frac{{\text{mA}}_{\text{Al}}-{\text{mA}}_{\text{m}}}{{\text{mA}}_{\text{Al}}}\\ \;=\frac{{\text{mAs}}_{\text{Al}}-{\text{mAs}}_{\text{m}}}{{\text{mAs}}_{\text{Al}}}\;=\;\frac{{\text{HU}}_{\text{Al}}-{\text{HU}}_{\text{m}}}{{\text{HU}}_{\text{Al}}}\\ \;=\frac{{\text{T}}_{\text{Al}}-{\text{T}}_{\text{m}}}{{\text{T}}_{\text{Al}}}\;=\;\frac{{\text{DOSE}}_{\text{Al }}-{\text{DOSE}}_{\text{m}}}{{\text{DOSE}}_{\text{Al }}} \end{array}$$

and

(9) $$\begin{array}{l} \frac{{\text{T}}_{\text{m}}}{{\text{T}}_{\text{Al}}}=\;\frac{{\text{DOSE}}_{\text{m}}}{{\text{DOSE}}_{\text{Al}}}\;=\;\frac{{\text{HU}}_{\text{Al}}}{{\text{HU}}_{\text{m}}}\\ \;\;=\;\frac{{\text{mAs}}_{\text{Al}}}{{\text{mAs}}_{\text{m}}}\;=\;\frac{{\text{mA}}_{\text{Al}}}{{\text{mA}}_{\text{m}}}\;=\frac{{\text{S}}_{\text{Al}}}{{\text{S}}_{\text{m}}}\\ \;=\;\text{1}\;+\;∆ \end{array}$$

where ∆ = λ_m_ t_Al_.

The values of λ_m_ resulting in best fit to Eq. (9) are reported in Table 1 of Okunade[6] for filter materials m with atomic numbers ranging from 12 to 39 and kVp ranging between 50 and 140 when reference filter is assumed to be aluminum.

From the simulated data and curve-fitting exercises carried out in this work,

(10) $$\frac{{\text{T}}_{\text{m}}}{{\text{T}}_{\text{Al}}}=\;\frac{{\left[{\text{CNR}}_{\text{m}}\right]}^{2}}{{\left[{\text{CNR}}_{\text{Al}}\right]}^{2}}\;=\;1\;+\;∆$$

Calculations carried out using copper as reference filter in comparison with filter material m resulted in equation of the form

(11) $$\frac{{\text{T}}_{\text{m}}}{{\text{T}}_{\text{Cu}}}=\;\frac{{\left[{\text{CNR}}_{\text{m}}\right]}^{\text{2}}}{{\left[{\text{CNR}}_{\text{Cu}}\right]}^{\text{2}}}\;=\;\text{1}\;+\;∆$$

where ∆= λ_m_ t_cu_

The values of λ_m_ resulting in best fit to Eq. (11) are reported in Table 2 of this text. Thus from Eqs. (5), (9) and (10), for *spectrally* equivalent beams emanating from filter material m and aluminum filter at ‘spectrally’ equivalent thicknesses respectively, FOM is of the form

**Table 2:** Values of λ_m_ resulting in best fit to Eq. (11). r^2^ gives the range of coefficient of regression

*Filter*	*50 kVp*	*60 kVp*	*70 kVp*	*80 kVp*	*90 kVp*	*100 kVp*	*110 kVp*	*120 kVp*	*130 kVp*	*140 kVp*	*r^2^*
^12^Mg	−1.407940	−1.428000	−1.432290	−1.420970	−1.414910	−1.403030	−1.394460	−1.400740	−1.372860	−1.383940	0.9939 - 0.9974
^13^Al	−1.097770	−1.128060	−1.135200	−1.133430	−1.125200	−1.118910	−1.111830	−1.102740	−1.095260	−1.092290	0.9971 - 0.9992
^14^Si	−0.854570	−0.904057	−0.905371	−0.920514	−0.919143	−0.919486	−0.914171	−0.910571	−0.905314	−0.899657	0.9958 - 0.9998
^23^V	−0.090857	−0.110857	−0.119143	−0.124571	−0.123943	−0.125257	−0.125714	−0.125943	−0.124971	−0.125886	0.9890 - 0.9999
^26^Fe	0.012800	−0.005086	−0.018514	−0.024515	−0.026800	−0.028343	−0.030229	−0.032000	−0.033029	−0.032972	0.8800 - 0.9957
^28^Ni	0.033372	0.021428	0.014514	0.010000	0.005714	0.004400	0.001930	0.000914	−0.000229	−0.000286	0.8843 - 0.9657
^30^Zn	0.033028	0.029257	0.026000	0.024343	0.023486	0.021943	0.021543	0.020571	0.020171	0.019658	0.9969 - 0.9994
^32^Ge	0.013829	0.026286	0.028857	0.029486	0.030686	0.031143	0.030972	0.031143	0.030686	0.030971	0.8721 - 0.9994
^39^Y	−0.010451	−0.030800	0.003257	0.026171	0.031771	0.036914	0.040971	0.045771	0.047086	0.061372	0.8998 - 0.9994

(12) $$\text{FOM}\;=\;\frac{{\left[{\text{CNR}}_{\text{m}}\right]}^{\text{2}}}{{\text{DOSE}}_{\text{m}}}\;=\;\frac{{\left[{\text{CNR}}_{\text{Al}}\right]}^{\text{2}}}{{\text{DOSE}}_{\text{Al}}}$$

and the CNR for beam transmitted through filter material m in comparison with that through a reference filter (aluminum) is of the form

(13) $${\text{CNR}}_{\text{m}}\;=\;{\text{CNR}}_{\text{Al}}\;\times \;\sqrt{1\;+\;∆}$$

In order to obtain spectral that is exactly equal in shape and size (or that will produce exactly the same values of transmittance, T, contrast, C, dose, DOSE, contrast-to-noise ratio, CNR, and figure-of-merit, FOM), the spectral data generated from filter material, say copper, in comparison with those from aluminum at ‘spectrally’ equivalent thickness have to be scaled across the entire energy spectrum by using the factor α. Mathematically, for this, we can write,

(14) $${ɸ}_{\mathrm{Al}}\left(E_{i}\right)\;=\;\alpha \;{ɸ}_{\mathrm{Cu}}\left(E_{i}\right)$$

where $\alpha =\frac{1}{1+∆}$

Note that the values of number of photons (per energy bin E_i_) transmitted by a filter with higher *Z*-value are greater than those transmitted by filter of lower *Z*-values when the tube is operated at the same kVp and tube loading. The use of this scaled spectral results in models of the form

(15) $$\begin{array}{l} {ɸ}_{\text{ref}}{\text{(E}}_{\text{i}}\text{)}\;=\;\alpha \times {ɸ}_{\text{m}}{\text{(E}}_{\text{i}}\text{)}\\ {\text{T}}_{\text{ref}}\;=\;\alpha \times {\text{T}}_{\text{m}}\\ {\text{DOSE}}_{\text{ref}}\;=\;\alpha \times {\text{DOSE}}_{\text{m}}\\ {\text{mAs}}_{\text{m}}\;=\;\alpha \times {\text{mAs}}_{\text{ref}}\\ {\text{mA}}_{\text{m}}\;=\;\alpha \times {\text{mA}}_{\text{ref}}\\ s_{\text{m}}\;=\;\alpha \times s_{\text{ref}}\\ {\text{HU}}_{\text{m}}\;=\;\alpha \times {\text{HU}}_{\text{ref}} \end{array}\}$$

The thickness of a given material, t_eq_, that is ‘spectrally’ equivalent to a given aluminum thickness t_Al_ is of the form[6]

(16) $${\text{t}}_{\text{eq}}={\text{t}}_{\text{Al}}\sum_{\text{n}=\text{0}}^{\text{n}=\text{4}}\omega_{\text{n}}{\text{V}}^{\text{n}}$$

The values of ω_n_ resulting in best fits to Eq. (16) when *Z*-value ranges between 12 and 39 are obtainable from Table 5 of Okunade.[6] When *Z*-values are 12, 13 and 14, the value of the thickness (t_eq_) of other filter material that is ‘spectrally’ equivalent to copper thickness t_cu_ can be obtained from equation of the form

(17) $${\text{t}}_{\text{eq}}\;=\;{\text{t}}_{\text{Cu}}\sum_{\text{n}=\text{0}}^{\text{n}=\text{4}}\kappa_{\text{n}}\;{\text{V}}^{\text{n}}$$

where κ_n_ is given by

(17a) $${\text{κ}}_{\text{n}}\;=\;\omega_{\text{n}}\;+\;\frac{\alpha_{\text{n}}}{{\text{t}}_{\text{Cu}}}$$

When *Z*-values are 23, 26, 28, 30, 32 and 39, the value of the thickness (t_eq_) of other filter material that is ‘spectrally’ equivalent to copper thickness t_cu_ can be obtained from equation of the form

(18) $${\text{t}}_{\text{eq}}\;=\;{\text{t}}_{\text{Cu}}\sum_{\text{n}=\text{0}}^{\text{n}=\text{4}}\omega_{\text{n}}{\text{V}}^{\text{n}}$$

The values of ω_n_ and α_n_ resulting in best fits to Eqs. (17) and (18) are shown in Table 3 of this text.

**Table 3:** Values of ω_n_ and α_n_ resulting in best fit to Eqs. (17-18). r^2^ is the coefficient of regression

*Filter*	ω_0_	ω_1_	ω_2_	ω_3_	ω_4_	*r^2^*
^12^Mg	60.5833	5.88075E−1	−7.96244E−3	4.67883E−5	−1.04474E−7	0.9949
^13^Al	32.2241	1.81886E−1	−2.16175E−3	1.07441E−5	−1.94637E−8	0.9966
^14^Si	24.2750	3.68141E−1	−5.12638E−3	3.18642E−5	−7.45273E−8	0.9969
^23^V	2.74584	1.02934E−2	−1.33379E−4	7.72564E−7	−1.66981E−9	0.9961
^26^Fe	1.44884	2.37010E−3	−2.12339E−5	7.56049E−8	−6.95810E−8	0.9950
^28^Ni	1.03401	8.21319E−4	−7.35967E−6	3.24672E−8	−5.83929E−11	0.9979
^30^Zn	1.10266	2.31597E−4	−1.50300E−6	2.09495E−9	9.63671E−12	0.9986
^32^Ge	1.40684	−3.33777E−3	4.75635E−5	−3.01819E−7	7.13212E−10	0.9920
^39^Y	1.18781	−1.02043E−2	1.33242E−4	−7.87023E−7	1.74605E−9	0.9989
*Filter*	α_0_	α_1_	α_2_	α_3_	α_4_	*r^2^*

^12^Mg	−2.86201E−2	−1.49630E−2	2.87569E−4	−2.01749E−6	5.21297E−9	0.9956
^13^Al	−1.70680E−1	1.80353E−3	−2.46925E−5	2.57028E−7	8.76419E−10	0.9951
^14^Si	1.83040E−1	−1.49477E−2	2.53103E−4	−1.75051E−6	4.39033E−9	0.9893

## Methods

### Simulations of beams of the same/close shape and size, contrast, contrast-to-noise ratio, dose and figure-of-merit

In order to simulate X-ray beams of the same/close shape and size (*spectrally* equivalent beams), calculations were carried out for the matching of hardening and attenuation from aluminum and copper filters (reference filters) and other filter materials that are different from aluminum and copper. The matching exercises involved the use of the numerical algorithms earlier reported by Jennings.[8] The thicknesses of aluminum filter used were between 1 and 6 mm at 1 mm increments while those of copper were between 0.05 and 0.3 mm at 0.05 mm increments. The incident X-ray spectrals on the filter materials were determined by using polynomial functions earlier reported by Boone and Seibert.[12] Unfiltered tungsten anode spectra ɸ_0_(E_i_) in units of photons/mm^2^ at energy E_i_ in keV were calculated for values of kVp ranging between 50 and 140 in 10 kVp steps.

**Figure 1:**
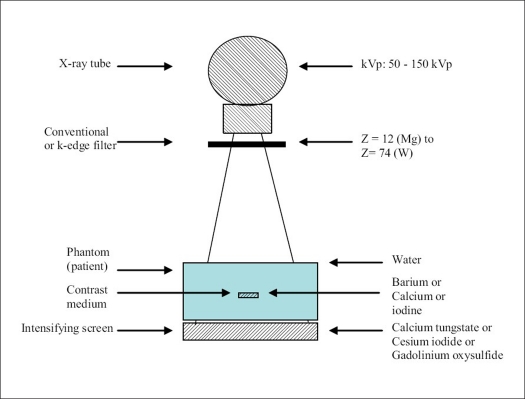
Schematic diagram for the implementation of the computer simulations

The transmission, contrast, contrast-to-noise ratio, figure-of-merit and dose were calculated using equations presented in the theory section above. A total of 10 values of kVp, 8 alternative conventional filter materials (other than aluminum/copper) and 6 reference filter thicknesses of aluminum/copper were considered. Simulations were carried out for the evaluation of T, C, CNR, FOM and DOSE for water phantom of thicknesses ranging between 5 and 30 cm at 5 cm increments. Also considered in these simulations are three contrast media - namely, calcium, barium and iodine; and three intensifying screens, which include CaWO_4_, CsI and Gd_2_O_2_S. By similar simulation exercise, the k-edge filter materials investigated in comparison with aluminum/copper filters include lanthanium, gadolinium, holmium, thulium and tungsten. The interaction data of photons for water (patient phantom) and those for elemental filters that were considered were taken from McMaster *et al*.,[13] while those for the contrast media and intensifying screens were from Hubbell and Seltzer.[14] All the models reported in this work were fitted using the curve-fit least-square routines implemented in the computer package GRAF4WIN.[15] Figure 1 shows the schematic diagram for the implementation of the computer simulations.

### Computer code EQSPECT.FOR

A FORTRAN source codes EQSPECT.FOR was developed for the execution of the simulations of same/close shape and size of beams, C, CNR, FOM and DOSE. In these computer codes, the algorithms reported[12] for the simulations of energy distribution of X-ray beams permit the specification of values of kVp other than those of multiples of 10 kV (say 86 kV). The interpolations for values of λ_m_ at values of kVp other than multiples of 10 kVp were carried out using cubic spline computer routines (named DEFSAL and DEFSCU) that were embedded in EQSPECT.FOR. A brief description of the main steps in the execution of EQSPECT.FOR is presented in Table 4.

**Table 4:** Main steps in the execution of the computer program EQSPECT.FOR^a^

The main steps in the execution of the software computer program, EQSPECT.FOR for the evaluation of performance characteristics of elemental filters materials (conventional and k-edge) in medical diagnostics are as follows:			
STEP 1:	Main program: Computes the spectral distribution for a specified operating potential (50-150 kVp) using the algorithms reported by Bonne and Siebert.[12]		
	Input: (1) kVp (2) ripple factor for X-ray tube; 100 (%) for single phase generators (%) (3) thickness of intensifying screen (mg/cm^2^) (4) thickness of contrast medium (mg/cm^2^) (5) type of comparison: spectrally equivalent thickness or arbitrary thickness (6) thickness of reference filter (mm) (7) thickness of alternative filter (if arbitrary) (mm) (8) atomic number of alternative filter.		
STEP 2	Call EQAL OR EQCU: Computes the thickness of a specified alternative filter material that will generate equivalent spectral (or produce equal hardening/shape) as a specified thickness of aluminum (EQAL) or copper (EQCU) filter.		
STEP 3:	Call MATCH:	(A)	Without scaling of spectral
			Compute the thickness of an alternative filter material that will produce same hardening as a specified thickness of an arbitrary reference filter material.
			Input data: (1) incident spectral (unfiltered spectral) (2) contrast medium (barium or calcium or iodine) (3) image receptor (assumed ideal, hence absorb all energy: calcium tungstate or cesium iodide or gadolinium oxysulfide).
			Output data: (1) kVp (2) filter thickness (3) ratios of fluence, exposure, kerma, dose, contrast, contrast-to-noise and figure-of-merit for the two filter materials (at ‘spectrally’ equivalent thicknesses. (3) α (4) δ (5) ‘spectrally’ equivalent thickness.
		(B)	Repeat (A) using the factor, α
		(C)	Output: Spectral distributions for (1) unfiltered beam (2) filtered beam (3) transmitted through reference filter (4) transmitted through alternative filter.

a The program and the 32 data files that are required for its execution are available for download via http://www4.webng.com/okunade or request via e-mail from the author.

**Table 5:** Comparison of the performance of filters relative to a 0.200-mm Cu filter at ‘spectrally’ equivalent thicknesses

(i) Aluminum: t_eq_ = 7.34 mm Al^a^												
		*Present work*								*Nagel[9]*		
			
		*With same exposure time*				*With increase in exposure time^b^*				*With increase in exposure time^b^*		
				
*kVp*	*t_eq_ (mm Al)*	$\frac{{\text{T}}_{\text{Cu}}}{{\text{T}}_{\text{Al}}}$	$\frac{{\text{DOSE}}_{\text{Cu}}}{{\text{DOSE}}_{\text{Al}}}$	$\frac{{\text{C}}_{\text{Cu}}}{{\text{C}}_{\text{Al}}}$	$\frac{{\text{FOM}}_{\text{Cu}}}{{\text{FOM}}_{\text{Al}}}$	$\frac{{\text{T}}_{\text{Cu}}}{{\text{T}}_{\text{Al}}}$	$\frac{{\text{DOSE}}_{\text{Cu}}}{{\text{DOSE}}_{\text{Al}}}$	$\frac{{\text{C}}_{\text{Cu}}}{{\text{C}}_{\text{Al}}}$	$\frac{{\text{FOM}}_{\text{Cu}}}{{\text{FOM}}_{\text{Al}}}$	$\frac{{\text{S}}_{\text{Cu}}}{{\text{S}}_{\text{Al}}}$	$\frac{{\text{DOSE}}_{\text{Cu}}}{{\text{DOSE}}_{\text{Al}}}$	$\frac{{\text{C}}_{\text{Cu}}}{{\text{C}}_{\text{Al}}}$
50	7.30 (7.31)	1.290	1.289	0.999	0.998	1.000	1.000	0.999	0.998	1.300	0.996	1.001
70	7.42 (7.42)	1.318	1.318	0.999	0.997	1.000	1.000	0.999	0.997	1.311	1.001	1.002
100	7.45 (7.45)	1.322	1.321	1.000	1.000	1.000	1.000	1.000	1.000	1.312	1.001	1.001
(ii) Iron: t_eq_ = 0.306 mm Fe^a^												
*kVp*	*t_eq_ (mm Fe)^c^*	$\frac{{\text{T}}_{\text{Cu}}}{{\text{T}}_{\text{Fe}}}$	$\frac{{\text{DOSE}}_{\text{Cu}}}{{\text{DOSE}}_{\text{Fe}}}$	$\frac{{\text{C}}_{\text{Cu}}}{{\text{C}}_{\text{Fe}}}$	$\frac{{\text{FOM}}_{\text{Cu}}}{{\text{FOM}}_{\text{Fe}}}$	$\frac{{\text{T}}_{\text{Cu}}}{{\text{T}}_{\text{Fe}}}$	$\frac{{\text{DOSE}}_{\text{Cu}}}{{\text{DOSE}}_{\text{Fe}}}$	$\frac{{\text{C}}_{\text{Cu}}}{{\text{C}}_{\text{Fe}}}$	$\frac{{\text{FOM}}_{\text{Cu}}}{{\text{FOM}}_{\text{Fe}}}$	$\frac{{\text{S}}_{\text{Cu}}}{{\text{S}}_{\text{Fe}}}$	$\frac{{\text{DOSE}}_{\text{Cu}}}{{\text{DOSE}}_{\text{Fe}}}$	$\frac{{\text{C}}_{\text{Cu}}}{{\text{C}}_{\text{Fe}}}$

50	0.303 (0.305)	0.992	0.992	1.000	0.999	1.000	1.000	1.000	0.999	1.000	0.997	1.000
70	0.305 (0.307)	1.000	1.000	0.999	0.999	1.000	1.000	0.999	0.999	1.002	1.000	1.001
100	0.307 (0.308)	1.004	1.004	0.999	0.999	1.000	1.000	0.999	0.999	1.003	1.000	1.001
(iii) Yttrium: t_eq_ = 0.18 mm Y^a^												
*kVp*	*t_eq_ (mm Y)^c^*	$\frac{{\text{T}}_{\text{Cu}}}{{\text{T}}_{\text{Y}}}$	$\frac{{\text{DOSE}}_{\text{Cu}}}{{\text{DOSE}}_{\text{Y}}}$	$\frac{{\text{C}}_{\text{Cu}}}{{\text{C}}_{\text{Y}}}$	$\frac{{\text{FOM}}_{\text{Cu}}}{{\text{FOM}}_{\text{Y}}}$	$\frac{{\text{T}}_{\text{Cu}}}{{\text{T}}_{\text{Y}}}$	$\frac{{\text{DOSE}}_{\text{Cu}}}{{\text{DOSE}}_{\text{Y}}}$	$\frac{{\text{C}}_{\text{Cu}}}{{\text{C}}_{\text{Y}}}$	$\frac{{\text{FOM}}_{\text{Cu}}}{{\text{FOM}}_{\text{Y}}}$	$\frac{{\text{S}}_{\text{Cu}}}{{\text{S}}_{\text{Y}}}$	$\frac{{\text{DOSM}}_{\text{Cu}}}{{\text{DOSM}}_{\text{Y}}}$	$\frac{{\text{C}}_{\text{Cu}}}{{\text{C}}_{\text{y}}}$

50	0.188 (0.184)	1.033	1.038	1.001	1.007	0.998	1.002	1.001	1.007	0.999	1.040	1.000
70	0.183 (0.180)	1.009	1.010	1.003	1.006	0.999	1.001	1.003	1.006	1.000	1.009	0.999
100	0.181 (0.177)	0.998	0.999	1.003	1.005	1.000	1.000	1.003	1.005	0.997	1.002	0.998

a From Table 5 of Nagel[9].

b Values obtained by using differential exposure time that will make the beams transmitted through pairs of filter materials to be of the same size (or intensity). Note that t_eq_ is slightly dependent on kVp. This increase in exposure time is for filter material with lower *Z*-value.

c Values without brackets are those obtained by using algorithm reported by Jennings[8], and those in brackets are those obtained by using Eqs. (17–18) in this text.

## Results

**Figure 2:**
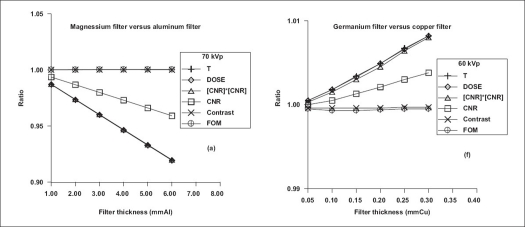
Ratios of T, DOSE, [CNR]^2^, CNR, contrast and FOM for beams transmitted through aluminum/copper filter (denominator) and selected alternative filter materials (numerator) at ‘spectrally’ equivalent thicknesses. This is for intensifying screen of 80 mg/cm^−2^ Gd_2_O_2_S, contrast medium of 10 mg/cm^−2^ iodine and object of 20 cm thick water phantom. The values of root-mean-square error are less than 1.0% for all cases of matching the hardening of pairs of conventional filters

**Figure 3:**
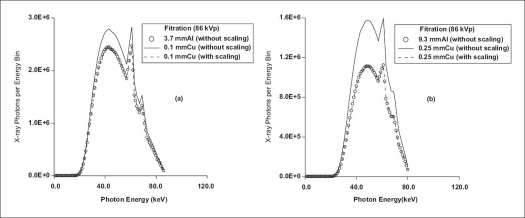
Comparison of the shape and size of transmitted spectral energy distribution at ‘spectrally’ equivalent thicknesses of aluminum and copper filter materials. The spectrals compared are (i) without scaling, Φ_Al_(E_i_) and Φ_Cu_(E_i_) and (ii) with scaling, Φ_Al_(E_i_) and aΦ_Cu_(E_i_)

**Figure 4:**
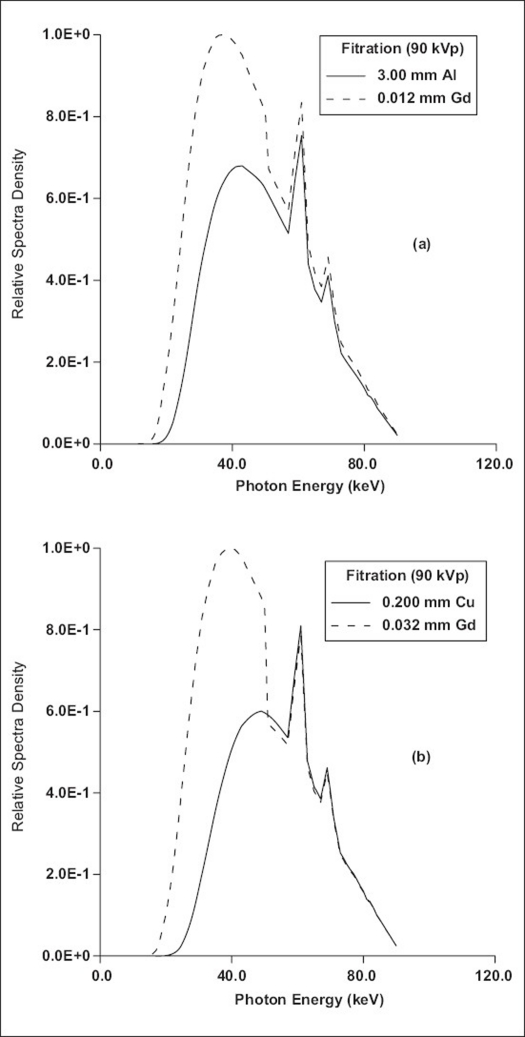
Comparison of the shape and size of transmitted spectral energy distribution at ‘spectrally’ equivalent thicknesses of aluminum/copper and gadolinium filters. The values of the minimum root-mean-square error in the matching of hardening were found to be 24.5% for these aluminum and gadolinium filters and 44.4% for these copper and gadolinium filters. The gadolinium filter transmits more photons at energies below its k-edge than aluminum and copper filters. There is a significant disparity in the hardening and attenuation properties of aluminum/copper (conventional filter) and those of gadolinium (k-edged filter)

**Figure 5:**
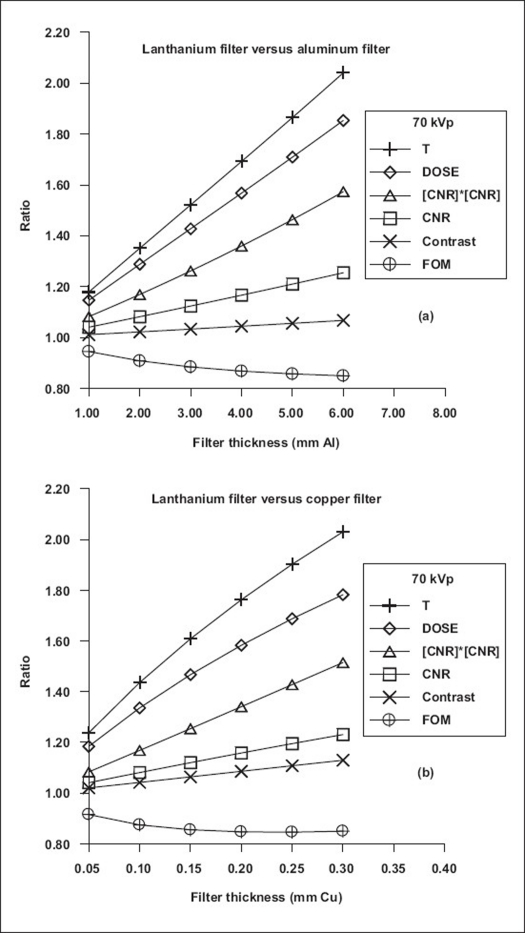
Ratios of T, DOSE, [CNR]^2^, CNR, contrast and FOM for beams transmitted through aluminum/copper filter (denominator) and lanthanium filter (numerator) at ‘spectrally’ equivalent thicknesses. This is for intensifying screen of 80 mg/cm_−2_ Gd_2_O_2_S, contrast medium of 10 mg/cm^−2^ iodine and object of 20 cm thick water phantom. There is a significant difference in both the shape and size of beams transmitted by aluminum/copper and lanthanium filters. Hence ratios of T, DOSE, [CNR]^2^, CNR, contrast and FOM deviate significantly from unity

Figure 2 shows the results of comparison of T, CNR, [CNR]^2^, C, DOSE and FOM for beams transmitted through aluminum/copper and selected alternative filter materials at ‘spectrally’ equivalent thicknesses. Specifically, for peak voltage of 86 kV, Figure 3 shows the results of comparison of the spectra transmitted through 3.7 mm Al and 9.3 mm Al and those through the respective spectrally equivalent thicknesses of copper, 0.1 mm Cu and 0.25 mm Cu, at equal tube loading. Table 5 shows the results of comparison of performance characteristics of other filter materials relative to copper filter. For the purpose of comparison with the work of Nagel,[9] these results are for 0.2 mm Cu filter, imaging of patient phantom of thickness 20 cm and contrast simulated with iodine (10 mg/cm^2^) and Gd_2_O_2_S intensifying screen (80 mg/cm^2^). Figure 4 shows the comparison of spectral shape and size obtained by forcing the same shape and size for aluminum/copper and gadolinium filters using the algorithms reported by Jennings.[8] The results of the comparison of transmission, contrast, CNR, [CNR^2^] DOSE and FOM obtained by the matching of spectral shape and size of beams transmitted by aluminum/copper and lanthanium are shown in Figure 5. To further provide verification of the validity of the formulations (Eqs. 8–15) presented in this work, the results of comparison carried out for 0.088 mm Gd filter and 0.097 mm Cu filter using EQSPECT.FOR are presented in Table 6.

**Table 6:** Performance characteristics of 0.097 mm Cu filter relative to 0.088 mm Gd filter

*kVp*	*Present work*		*Exposure time ratio Nagel*[9]
		
	$\frac{{\text{T}}_{\text{Gd}}}{{\text{T}}_{\text{Cu}}}$	$\frac{{\text{DOSM}}_{\text{Gd}}}{{\text{DOSM}}_{\text{Cu}}}$	$\frac{{\text{S}}_{\text{Cu}}}{{\text{S}}_{\text{Gd}}}$
60	1.17	1.18	1.20
80	1.33	1.36	1.39
100	1.39	1.42	1.44

## Discussion

The value of CNR for the beam filtered by 0.1 mm Cu exceeds that of the beam filtered by 3.7 mm Al by 7.0% for the imaging of a 20-cm patient phantom with the contrast simulated with iodine (12 mg/cm^2^) and CsI intensifying screen (60 mg/cm^2^). For this same imaging, the value of CNR for beam filtered by 0.25 mm Cu exceeds that for beam filtered by 9.3 mm Al by 19.0%. For the case of 3.7 mm Al and 0.10 mm Cu filtrations, the values of T_Cu_, DOSE_Cu_ and [CNR_Cu_]^2^ exceed those of T_Al_, DOSE_Al_ and [CNR_Al_]^2^ by 14.5% respectively while C_Cu_ ≅ C_Al_ and FOM_Cu_ ≅ FOM_Al_. This is in good agreement with the result from earlier work,[8] which reported a surplus of 14.8% for T_Cu_ over T_Al_. At spectrally equivalent thicknesses, the difference in exposure time obtained experimentally for these aluminum and copper filters to produce beam of exactly the same shape and size was 15.6%, with the former filter requiring more exposure time.[8] This is in reasonable agreement with the theoretical formulations [Eqs. (8) and (9) of this text], which yield 14.5% as the value of ∆ × 100% (where ∆ = λ_m_ t_ref_). With the beams from these filters (copper and aluminum at ‘spectrally’ equivalent thicknesses) numerically made exactly equal in shape and size {ɸ_Al_(E_i_) = αɸ_Cu_(E_i_)}, the results obtained by simulations were such that T_Cu_ ≅ T_Al_, DOSE_Cu_ ≅ DOSE_Al_, CNR_Cu_ ≅ CNR_Al_, [CNR_Cu_]^2^ ≅ [CNR_Al_]^2^, C_Cu_ ≅ C_Al_ and FOM_Cu_ ≅ FOM_Al_.

In the case of beam filtered by 9.3 mm Al in comparison with that filtered by 0.25 mm Cu and for spectral without scaling (same mAs and heat capacity), the values of T_Cu_, DOSE_Cu_ and [CNR_Cu_]^2^ exceed those of T_Al_, DOSE_Al_ and [CNR_Al_]^2^ by 41.9% respectively while C_Cu_ ≅ C_Al_ and FOM_Cu_ ≅ FOM_Al_. Theoretical result presented[8] shows this surplus value to be 41.6%. With the transmitted spectrum through the copper filter theoretically scaled {ɸ_Al_(E_i_ = αɸ_Cu_ (E_i_)} in comparison with aluminum filter, the results obtained by simulations were such that T_Cu_ ≅ T_Al_, DOSE_Cu_ ≅ DOSE_Al_, [CNR_Cu_]^2^ ≅ [CNR_Al_]^2^, CNR_Cu_ ≅ CNR_Al_, C_Cu_ ≅ C_Al_ and FOM_Cu_ ≅ FOM_Al_.

Results of spectrometric measurements carried out by Jennings[8] show that 4.08 mmAl, 0.11 mmCu and 0.10 mm Y transmit *spectrally* equivalent beams. For 4.08 mm Al in comparison with 0.11 mm Cu and without the scaling of spectral (same mAs and heat capacity), using Eqs. (8–11), the values of T_Cu_, DOSE_Cu_ and [CNR_Cu_]^2^ exceed those of T_Al_, DOSE_Al_ and [CNR_Al_]^2^ by 16.2% respectively while C_Cu_ ≅ C_Al_ and FOM_Cu_ ≅ FOM_Al_. The same surplus was found for yttrium in comparison with aluminum at the respective ‘spectrally’ equivalent thicknesses of 0.10 mm Y and 4.08 mm Al while C_Y_ ≅ C_Al_ and FOM_Y_ ≅ FOM_Al_. This is in good agreement with the results from the work of Jennings,[8] which reported a difference of 15.3% (experimental) and 16.7% (theoretical) for T_Cu_ and T_Al_ with copper transmitting surplus. Also, reported by the same author[8] was a difference of 15.4% (experimental) and 16.4% (theoretical) for T_Y_ and T_Al_ with yttrium filter transmitting surplus. With the transmitted spectrum through the copper filter scaled (mAs and heat capacity scaled by the factor α), the results obtained from the simulations carried out in this work were such that T_Al_ ≅ T_Cu_ ≅ T_Y_, DOSE_Al_ ≅ DOSE_Cu_ ≅ DOSE_Y_, [CNR_Al_]^2^ ≅ [CNR_Cu_]^2^ ≅ [CNR_Y_]^2^, C_Al_ ≅ C_Cu_ ≅ C_Y_ and FOM_Al_ ≅ FOM_Cu_ ≅ FOM_Y_.

Using scaled spectrum {α Φ_Cu_ (E_i_)}, operating the tube with copper filter at reduced tube loading/heat capacity (α × mAs_Al_ or 1.35α × mAs_Al_ × kVp) results in same value of T, DOSE, C, CNR and FOM when compared with operating the tube with aluminum filter at higher tube loading/heat capacity (mAs_Al_ or 1.35 × mAs_Al_ × kVp). Note that whether the beams are scaled or not scaled, once the spectral shape is the same, the values of contrast and FOM will be the same. However, beams filtered by materials with higher values of atomic numbers are more intense (bigger in shape) and produce higher values of CNR and dose to patient than those filtered by material of lower atomic numbers at ‘spectrally’ equivalent thicknesses when the tube is operated at the same values of kVp, tube loading and heat capacity [Figures 2 and 3]. For a given filter material when compared with aluminum/copper at ‘spectrally’ equivalent thicknesses, only the shapes of transmitted spectra are the same. Depending on the *Z*-value, the sizes are not the same when the tube is operated at same values of kVp, tube loading and heat capacity [Figure 3]. At respective ‘spectrally’ equivalent thicknesses, the benefits derivable from the use of higher *Z*-value filter material in comparison with those from the use of lower *Z*-value include higher intensity, lower filter-generated scattered radiation and shorter tube current or exposure time. This can translate into an increase in the lifespan of tube (more filament hours by operating at reduced mAs) and reduction in the cost of dealing with the detrimental effects of heat and scattered radiation. Also, extra images could be obtained using the gain in exposure time and tube loading/heat capacity.

The spectrum obtained by numerically scaling the values of αɸ_Cu_ (E_i_) using the factor α (Eq. 14) is in agreement with that transmitted by 3.7 mm Al [Figure 3]. The scaled spectrum obtained from copper filter {αɸ_Cu_(E_i_)} and that directly obtained from aluminum {ɸ_Al_(E_i_)} were found to produce the same contrast, CNR, DOSE and FOM. Though not carried out in this work, experimentally at the same kVp, the spectra obtained from filter material with higher *Z*-value (say copper) in comparison with those with lower *Z*-value (say aluminum) could be obtained at the scaled intensity and dose (scaled to that obtainable from aluminum filter) by operating the tube at scaled tube loading and heat capacity. This scaling factor provides for the means of obtaining strictly equivalent (same shape and size) spectra from two different elemental filter materials (conventional filters) at the same value of kVp [Figure 3].

The validity of Eq. (15) had been reported in Okunade[6] using the case of peak voltage of 86 kV and comparison of 0.10 mm Cu with the corresponding ‘spectrally’ equivalent aluminum thickness, 3.7 mm Al (reference filter). Also, Figure 3 shows that the formulation presented as T_Al_ = α × T_Cu_ is valid. Hence all the formulations in Eq. (15) are valid. Table 5 shows that the values of ‘spectrally’ equivalent thicknesses obtained using Eqs.(16–18) are in good agreement with those earlier reported in literature.[8–9] Apart from providing comparison for aluminum/copper and any given filter material irrespective of atomic number, EQSPECT.FOR can be used to obtain relative performance for any arbitrary pair of filter materials, say silicon and iron. This is accomplishable by specifying the reference filter material as silicon and the alternative filter material as iron.

Generally, it is well known that it is not possible to have a complete spectral matching for conventional and k-edge filter materials. The k-edge discontinuity interferes substantially in the range of useful energies, resulting in beams with significant difference in shape and size. Unlike the case of the comparison of conventional filter materials (aluminum/copper) with other conventional filter materials, large values of the root-mean-square errors[8] were obtained when conventional filter materials were compared with k-edge filter materials. This implies a large difference between the actual transmission of aluminum/copper and the scaled transmission of the k-edge filters. It is not possible to derive simple mathematical formulations of the form with factors α, δ and ∆ for the comparison of performance of beams from conventional filter materials with those from k-edge filter materials since spectrals transmitted by these pair of filter materials differ in quality and quantity [Figure 4]. Theoretically, the transmission below the k-edge in terms of quality and quantity could not be matched in these comparisons. However, using Eq. (9), the results presented in Table 6 for the comparison of the overall quantity of photons in terms of the ratios of T and DOSE from beams transmitted by 0.097 mm Cu and 0.088 mm Gd are in reasonable agreement with experimentally measured ratio of exposure time reported by Nagel.[9]

## Conclusions

Models and software program have been presented for the comparative evaluation of the performance characteristics (contrast, contrast-to-noise ratio and FOM) of beams transmitted at spectral equivalent thicknesses by elemental filter materials (conventional and k-edge filters). Under this condition of spectral equivalence, the mathematical formulations presented show that when compared with one of lower *Z*-value, conventional filter material with higher *Z*-value offers opportunity for extended exposure time. This extended exposure time can translate into acquiring extra images using the gain in exposure time, tube loading and heat capacity. The computer software EQSPECT.FOR developed in this work can be used as a tool for the comparative investigation of filter materials with a view to determine alternative beams that could yield optimum performance in clinical radiological practice. The results from this comparative investigation can aid in the design of X-ray machines in such a way as to produce a reference shape and size of X-ray beam with which others can be compared. This could assist in the achievement of some degree of uniformity in clinical X-ray diagnostic practice.

## References

[1] Boone JM (1986). Equivalent spectra as an index of beam quality. Med Phys.

[2] Boone JM (1988). Three parameter equivalent spectra as an index of beam quality. Med Phys.

[3] Thoraeus R (1932). The study of the ionization method for measuring the intensity and absorption of Roentgen rays and efficiency of different filters used in therapy. Acta Radiol.

[4] Thoraeus R (1934). Tin filters in roentgen therapy. Acta Radiol.

[5] Okunade AA (2002). Numerical models for comparing filter materials for diagnostic radiology. Radiat Phys Isot.

[6] Okunade AA (2005). Parameterized algorithm for quantitative differentials in *spectrally* equivalent medical diagnostic x-ray beams. Med Phys.

[7] Nagel HD (1986). Aluminum equivalence of materials used in diagnostic radiology and its dependence on beam quality. Phys Med Biol.

[8] Jennings RJ (1988). A method for comparing beam-hardening filter materials for diagnostic radiology. Med Phys.

[9] Nagel HD (1989). Comparison of the performance of characteristics of conventional and k-edge filters in general diagnostic radiology. Phys Med Biol.

[10] Birch R, Marshall M, Ardran GM (1979). Catalogue of spectral data for diagnostic x-rays. The Hospital Physicist’s Association Scientific Report Series -30.

[11] Okunade AA (2005). Effective dose as a limiting quantity for the evaluation of primary barriers for diagnostic x-ray facilities. Health Phys.

[12] Boone JM, Seibert JA (1997). An accurate method for computer-generated tungsten anode x-ray spectra from 30 to 140 kV. Med Phys.

[13] McMaster WH, Del Grande NK, Mallett JH, Hubbell JH (1969). Compilation of X-ray cross section”.

[14] Hubbell JH, Seltzer SM (1995). Tables of x-ray mass attenuation coefficients and mass energy-absorption coefficients 1 keV to 20 MeV for elements 1-92 and 48 additional substances of dosimetric interest. National Institute of Standard and Technology Internal Report NISTIR.

[15] GRAF4WIN (1993-1994). A computer program for graphics and curve fitting GRAPHER(™) for Windows v1.2 Golden Software Incorporated.

